# Influence of cognitive reserve on technology use behavior in elderly people: The role of education and daily activities

**DOI:** 10.1002/alz70858_106866

**Published:** 2025-12-26

**Authors:** Elsa Araceli Revollo Sarmiento, Leticia Vivas, Deisy Krzemien

**Affiliations:** ^1^ Institute of Basic and Applied Psychology and Technology (IPSIBAT), National University of Mar del Plata (UNMDP), Psychology Faculty, Mar del Plata, Buenos Aires, Argentina

## Abstract

**Background:**

The connection between cognitive reserve (CR) and technology‐related behavior in elderly people is emerging as a relevant area of study in aging and gerontechnology research. Recent studies suggest that a higher level of cognitive reserve is associated with greater adaptability and openness to acquiring new skills, which facilitates the incorporation of technology into daily life (Pérez & Gomez, 2024). This study examines how cognitive reserve influences older adults' behavior regarding technology use.

**Method:**

79 cognitively healthy elderly people (77% women) participated in the study. The sample had a mean age of 70.64 (range 65‐89) and an average of 11.92 years of education (range 6‐17). A cross‐sectional correlational study was conducted. The variables analyzed included behavior regarding mobile phone use based on the SAMCOP model (Ma et al., 2016) and the categories of the cognitive reserve model (Rami et al., 2011).

**Result:**

A linear regression analysis was conducted to evaluate the relationship between mobile phone use behavior and the categories of cognitive reserve (activities of daily living, hobbies, education, and social life). The results indicate that education has a direct and significant influence on older adults' behavior towards mobile phone use (*p* < 0.01). Similarly, activities of daily living and social life show a significant trend of influence (*p* < 0.05). In contrast, the hobbies variable did not show a relevant effect on this behavior (*p* = 0.775).

**Conclusion:**

The analysis of the relationship between cognitive reserve and mobile phone use behavior in older adults reveals that factors such as education and activities of daily living and social life influence their adaptation to technology. In particular, academic education had a direct and strong impact on mobile phone use behavior, highlighting the importance of continuous education in active aging and technological integration. Although activities of daily living and social life showed a significant trend, hobbies did not appear to have a relevant influence on this behavior. These findings underscore the importance of cognitive reserve in promoting technology use among older adults and suggest areas of intervention to improve their digital inclusion.

